# Genome-wide identification and expression analysis of the *SPL* transcription factor family and its response to abiotic stress in Quinoa (*Chenopodium quinoa*)

**DOI:** 10.1186/s12864-022-08977-9

**Published:** 2022-11-25

**Authors:** Yanyan Ren, Rui Ma, Yue Fan, Bingjie Zhao, Peng Cheng, Yu Fan, Baotong Wang

**Affiliations:** 1 State Key Laboratory of Crop Stress Biology for Arid Areas, College of Plant Protection, Northwest A&F University, Yangling, 712100 Shaanxi People’s Republic of China; 2 College of Food Science and Engineering, Xinjiang Institute of Technology, 843100 Aksu, P.R. China; 3 School of Food and Biological Engineering, Chengdu University, Longquanyi District, 610106 Chengdu, P.R. China

**Keywords:** *Chenopodium quinoa*, *SPL* gene family, Genome-wide analysis, Abiotic stress

## Abstract

**Background:**

Squamous promoter binding protein-like (SPL) proteins are a class of transcription factors that play essential roles in plant growth and development, signal transduction, and responses to biotic and abiotic stresses. The rapid development of whole genome sequencing has enabled the identification and characterization of *SPL* gene families in many plant species, but to date this has not been performed in quinoa (*Chenopodium quinoa*).

**Results:**

This study identified 23 *SPL* genes in quinoa, which were unevenly distributed on 18 quinoa chromosomes. Quinoa *SPL* genes were then classified into eight subfamilies based on homology to *Arabidopsis thaliana SPL* genes. We selected three dicotyledonous and monocotyledonous representative species, each associated with *C. quinoa*, for comparative sympatric mapping to better understand the evolution of the developmental mechanisms of the *CqSPL* family. Furthermore, we also used 15 representative genes from eight subfamilies to characterize *CqSPL*s gene expression in different tissues and at different fruit developmental stages under six different abiotic stress conditions.

**Conclusions:**

This study, the first to identify and characterize *SPL* genes in quinoa, reported that *CqSPL* genes, especially *CqSPL1*, play a critical role in quinoa development and in its response to various abiotic stresses.

**Supplementary Information:**

The online version contains supplementary material available at 10.1186/s12864-022-08977-9.

## Background

Quinoa (*Chenopodium quinoa* Willd.), a halophytic pseudocereal crop (2n = 4 ×  = 36), originates from the Andean region of South America and generally grows on plateaus above 4500 m. Consequently, it is highly tolerant to abiotic stresses including drought, excess soil salinity, and frost [1, 2]. Quinoa kernels are alkaline, and have higher protein, vitamin, and mineral content than any traditional grain crop [3, 4]. Moreover, due to its potential health benefits the United Nations declared the year 2013 to be the International Year of Quinoa [5]. Furthermore, the publication of the quinoa genome has laid the foundation for quinoa genetic improvement and selective breeding [6, 7].

Transcription factors (TFs) are DNA-binding proteins that specifically interact with cis-acting elements in eukaryotic genomes, and are involved in almost all plant biological processes [8]. When plants experience biotic or abiotic stress, transcription factors bind to specific regions in gene promoters to activate or inhibit the transcription of downstream target genes that mediate defensive responses [9, 10]. The SPLs (SQUAMOSA-PROMOTER BINDING PROTEIN-LIKE) are a plant-specific family of TFs that bind to the SQUAMOSA-promoter [11, 12]. Each SPL contains a 76-residue SQUAMOSA-promoter binding protein (SBP) domain, two specific zinc finger motifs (Cys-Cys-His-Cys and Cys-Cys-Cys-His), and a nuclear localization signal (NLS) motif in the C-terminal region. Four residues in the SBP domain coordinate a zinc ion to maintain protein stability, while the NLS motif overlaps with a second zinc finger structure to guide proteins to the nucleus, thereby modulating the transcription of downstream genes [12–14].

Many *SPL* gene families have been identified in numerous plant species, including *Arabidopsis thaliana* [15, 16], *Salvia miltiorrhiza* [17], *Capsicum annuum* L. [18], *Zea mays* L. [19], *Ricinus communis* L. [20], *Malus domestica* [21], *Vitis vinifera* [22], *Glycine max* [23], *Solanum lycopersicum* [24], *Tartary buckwheat* [25], *Triticum aestivum* [26], and *Gossypium spp.* [27]. Huijser et al*.* identified and cloned the first two *SPL* genes, including the conserved structural MADS-BOX domain, in *Antirrhinum majus* [28]. These two genes were subsequently named *SPL1* and *SPL2* by Klein (1992) [12] and were shown to regulate flower development. To date, 16 *SPL* genes have been identified in *A. thaliana*, and these have been classified into eight groups based on their conserved SBP structural domain. These groups include *AtSPL7* (group I), *AtSPL1/12/14/16* (group II), *AtSPL8* (group III), *AtSPL6* (group IV), *AtSPL2/10/11* (group V), *AtSPL3/4/5* (group VI), *AtSPL13* (group VII), and *AtSPL9/15* (group VII). In Arabidopsis, *SPL* family genes have been shown to play significant roles in leaf, stem, and flower development [29, 30]. Furthermore, numerous studies of other plant species have demonstrated that *SPL* family genes also regulate various physiological aspects related to plant growth and development, including flower and fruit formation, stress response, and plant phase transition [31–34]. However, despite the identification of *SPL* genes in many plant species, their function remains poorly understood in *C. quinoa* [35, 36].

This study uses a recently published genome assembly to identify *SPL* genes in *C. quinoa* and to determine their structure, motif composition, chromosomal location, and whether or not they have undergone duplication [6]. We also evaluated the evolutionary relationships between *C. quinoa* and *A. thaliana*, *S. lycopersicum*, *V. vinifera*, *S. bicolor*, *O. sativa*, and *Z. mays* to understand the degree of conservation in the roles played by *SPL* genes in plant developmental processes. This study provides valuable information for screening important *SPL* genes in quinoa under various development conditions and offers a new theoretical basis for the functional analysis of the *SPL* gene family in other species.

## Results

### Identification of SPL genes in C. quinoa

A total of 23 *CqSPL* genes were identified in quinoa using two BLAST methods. These were named *CqSPL1*-*CqSPL23* based on their chromosome number (Additional file 2: Table S1). The general characteristics of all *CqSPL*s, including coding sequence length, molecular weight (MW), isoelectric point (pI), and subcellular localization, were determined using CELLO version 2.5 (http://cello.life.nctu.edu.tw/).

Among the 23 CqSPL proteins, CqSPL11 and CqSPL12 were the smallest with each containing only 119 amino acids. In contrast, CqSPL17 was the largest, and contained 1190 amino acids. Protein molecular mass ranged from 21.3 kDa (CqSPL12) to 132.135 kDa (CqSPL17), and pI values ranged from 5.74 (CqSPL15) to 10.24 (CqSPL1 and CqSPL12), with a mean of 6.69. We also found that four of the 23 CqSPL proteins contained the ANK domain. Subcellular localization results showed that all CqSPL proteins were located in the nucleus, with seven also present in the endoplasmic reticulum, eight in the cytoplasm and plasmid, nine in the chloroplast, and one (CqSPL9) in the plasmid (Table S1). We also found that *C. quinoa* contained more *SPL* genes (23) than *A. thaliana* (15), *S. lycopersicum* (15), *V. vinifera* (17), or *S. bicolor* (19), but less than *O. sativa* and *Z. mays*, each of which has 29 *SPL* genes [37–40].

### Multiple sequence alignment, phylogenetic analysis, and classification of CqSPL proteins

**Fig. 1:**
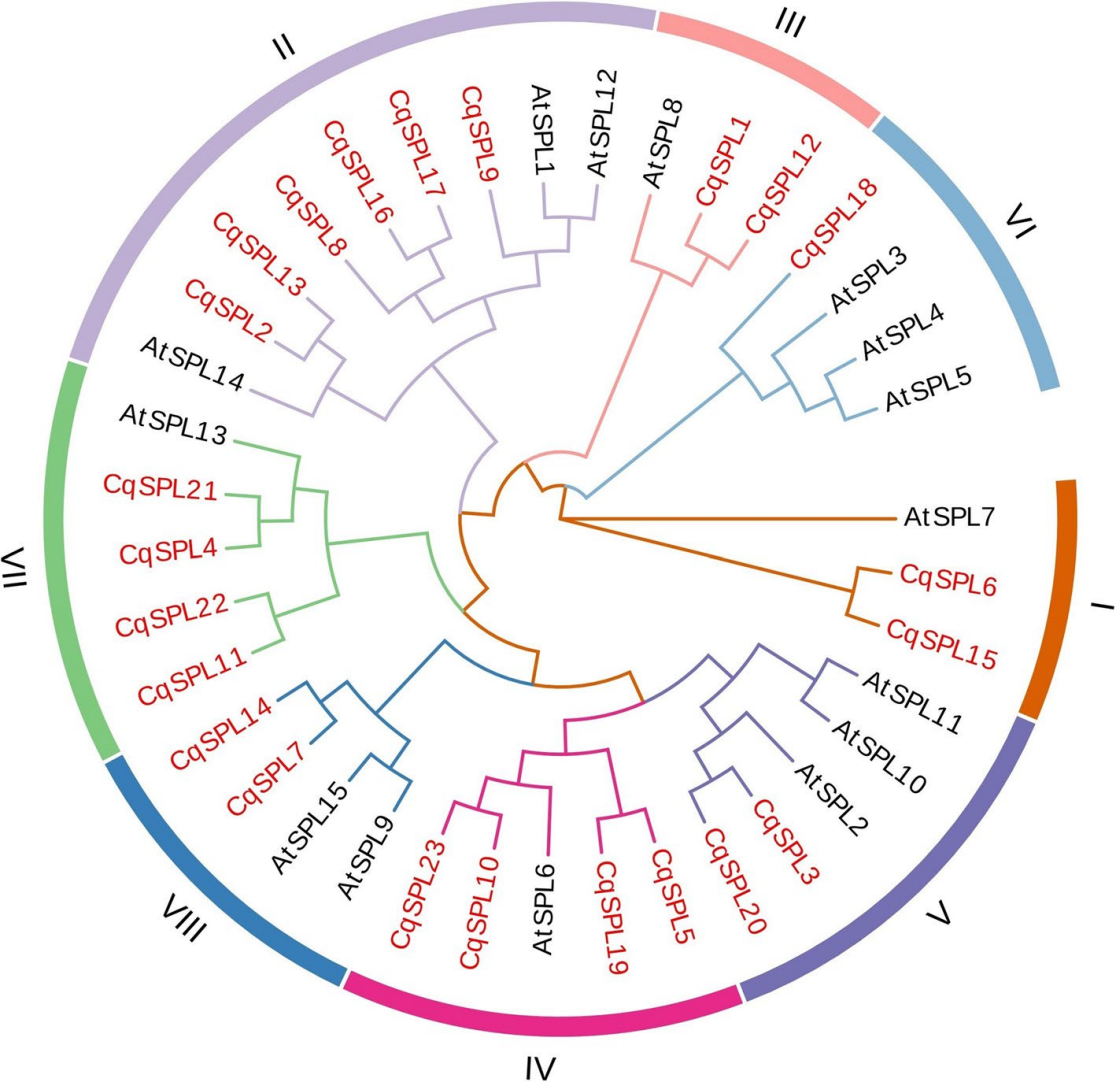
A phylogenetic tree of SPL proteins from Chenopodium quinoa and Arabidopsis thaliana constructed using MEGA 7.0. The tree shows the division of SPL proteins into eight subfamilies. SPL proteins from C. quinoa are labeled in red and SPL proteins from A. thaliana are labelled in black

The 23 CqSPL proteins were then divided into eight phylogenetic clades (groups 1–8) based on the previously proposed classification method. Their consensus with the classification groups of *Arabidopsis* SPL proteins suggests that *SPL* genes are strongly conserved during molecular evolution (Fig. 1; Additional file 2: Table S1).

Among the eight subfamilies, subfamily II had the most members (6 CqSPLs), while subfamily VI contained only one CqSPL. Subfamilies I, III, V, and VIII had two *CqSPL* genes each, and subfamilies IV and VII each contained four CqSPLs. The phylogenetic tree also showed that some CqSPLs clustered closely with AtSPLs (bootstrap support ≥ 70) (Fig. 1), which suggests that these proteins might be orthologous and therefore may possess similar biological functions.

### Multiple sequence alignment of AtSPLs with the eight CqSPL subfamilies

**Fig. 2:**
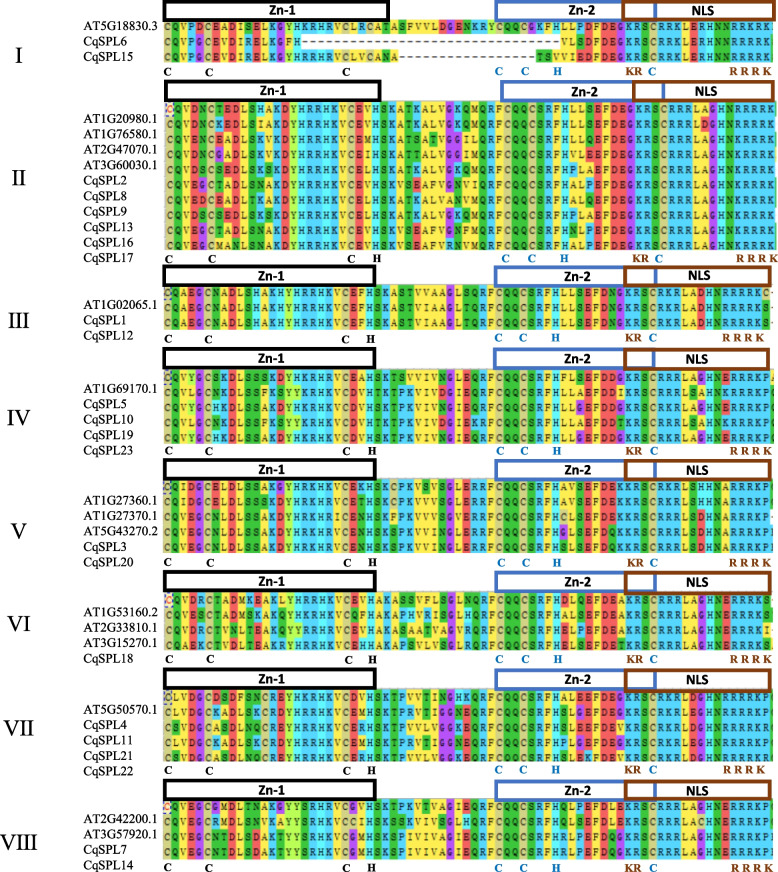
Multiple sequence alignment of SBP domains of eight subfamilies of the CqSPL protein family. The Zn-finger (Zn-1, Cys3His; Zn-2, Cys2HisCys) and NLS structures are indicated

Previous studies have reported that all SPL proteins contain conserved SBP domains. This includes two zinc fingers (Zn 1 and 2) and a bipartite nuclear localization signal (NLS) motif. The basic region consists of 14 conserved amino acids in a span of 70–80 amino acids (Fig. 2, Table S1). In the present study, only subfamily I was found to be not fully conserved between *C. quinoa* and *Arabidopsis*. The Zn-1 (Cys3His-type) finger of *CqSPL6* (subfamily I) lacked a Cys residue, and the Zn-2 (Cys2HisCys-type) finger from the same protein lacked C2H; these sequences are still conserved in *Arabidopsis* (Fig. 2). Conversely, the NLS motif was relatively conserved in quinoa but contains a mutation in one of the R’s in the RRRK sequence located at the C-terminus of the SBP domain in *Arabidopsis*. Finally, we found that the SBP domains of *Arabidopsis* and *C. quinoa* were very alike and therefore highly conserved, which suggests that the SBP structural domain was established at an early stage in plants.

### Conserved motifs and structural analysis of CqSPL genes

**Fig. 3:**
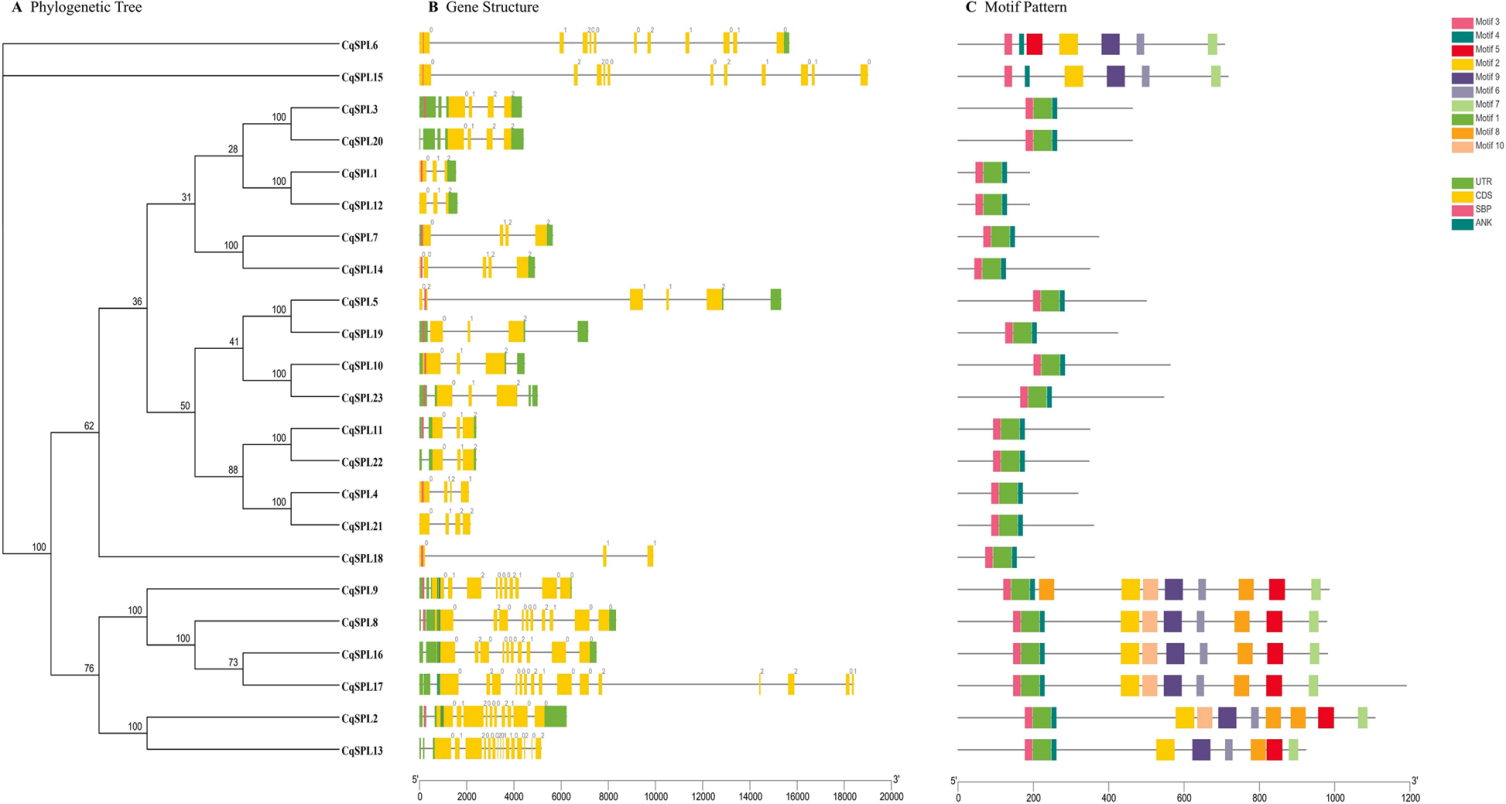
Analysis of conserved motifs and gene structures in the phylogenetic tree of 23 CqSPL genes. **A** A phylogenetic tree was constructed using the amino acid sequences of the quinoa SPL genes using the NJ method. **B** Exons and introns are shown as yellow rectangles and gray lines, respectively. The SBP conserved domain and ANK region are clearly marked. 0, 1, and 2 indicate exon phase. **C** Ten conserved motifs predicted in SPL proteins are shown as differently colored boxes

The exons and introns of *CqSPL* genes were identified by comparing them with their corresponding genomic DNA sequences. These results revealed that the 23 *CqSPL* genes contained different numbers of exons, ranging from 3 to 17. We also found that the SBP domain was present in most (17 or ~ 69.5%) *CqSPL* genes (Fig. 3, Additional files 2 and 3: Tables S1 and S2). Furthermore, *CqSPL1*, *CqSPL12*, and *CqSPL18* showed identical intron and exon structures, each containing three exons and two introns each (Fig. 3B). Six *CqSPL* genes had four introns, while *CqSPL13* and *CqSPL17*, both of which belong to subfamily II, had the most introns (16) (Fig. 3A, B). Generally, we found that *CqSPL* genes from the same subfamily had similar gene structures, but subfamily II showed greater differences in the number of introns. This may be due to evolution for more diverse functional roles.

Further structural analysis of *CqSPL* genes identified ten diverse motifs (denoted motifs 1–10). As shown in Fig. 3C, motifs 3 and 4 were widely distributed and were located adjacent to each other in the *CqSPL*s. *CqSPL* genes from the same subfamily usually possessed similar motif compositions. For instance, subfamily I genes contained motifs 2, 3, 4, 6, 7, and 9 (except for *CqSPL13*), while subfamily II contained all motifs (1–10). We also found that subfamilies III, IV, V, VI, VII, and VIII all contained the same motifs (1, 3, and 4). Furthermore, some motifs were found only in specific positions. For example, motifs 3 and 7 were always found at the start and the end of the series of unique motifs, while motif 1 was always located between motifs 3 and 4 in subfamily I (Fig. 3C, Table S2). In general, we found that genes from the same subfamily had similar structural compositions and clustered together, a finding that was consistent with the classification based on the phylogenetic tree.

### Chromosomal distribution and gene duplication of CqSPL genes

**Fig. 4:**
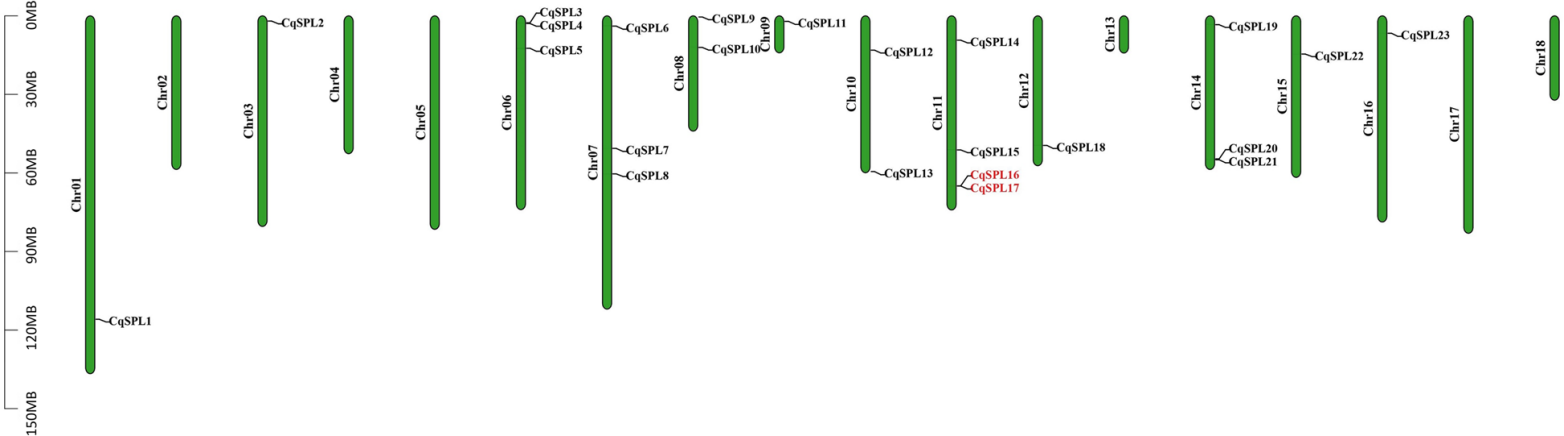
The distribution of 23 CqSPL genes on different chromosomes. The leftmost scale represents chromosome length. Green bars indicate chromosomes and to the left of each green bar is the chromosome number

Using the latest genome database, our analysis of the chromosomal localization of *SPL* genes demonstrated that the 23 *CqSPL* genes were unevenly distributed on chromosomes (Chr)1 to 18 (Fig. 4, Additional file 4: Table S3). Each *SPL* gene was named based on its physical location on chromosomes (Chr) 1 to 18. Conversely, *CqSPL* genes were not found on Chr2, Chr4, Chr5, Chr13, Chr17, and Chr18. In addition, we also found that Chr11 contained the most *CqSPL* genes (four or ~ 17.39% of the total), followed by Chr6, Chr7, and Chr14, which contained three (~ 13.04%) and Chr8 and Chr10, which both contained two (~ 8.70%) *CqSPL* genes. Finally, Chr1, Chr3, Chr9, Chr12, Chr15, and Ch16 each contained a single *CqSPL* gene (~ 4.35%). Almost all *SPL* genes were distributed at one of the ends of the 23 chromosomes; however Chr7 was an exception. Only one *SPL* gene duplication event was evident in *C. quinoa*, which featured *CqSPL16* and *CqSPL17* on Chr 11 ​(Fig. 4, Table S3).

**Fig. 5:**
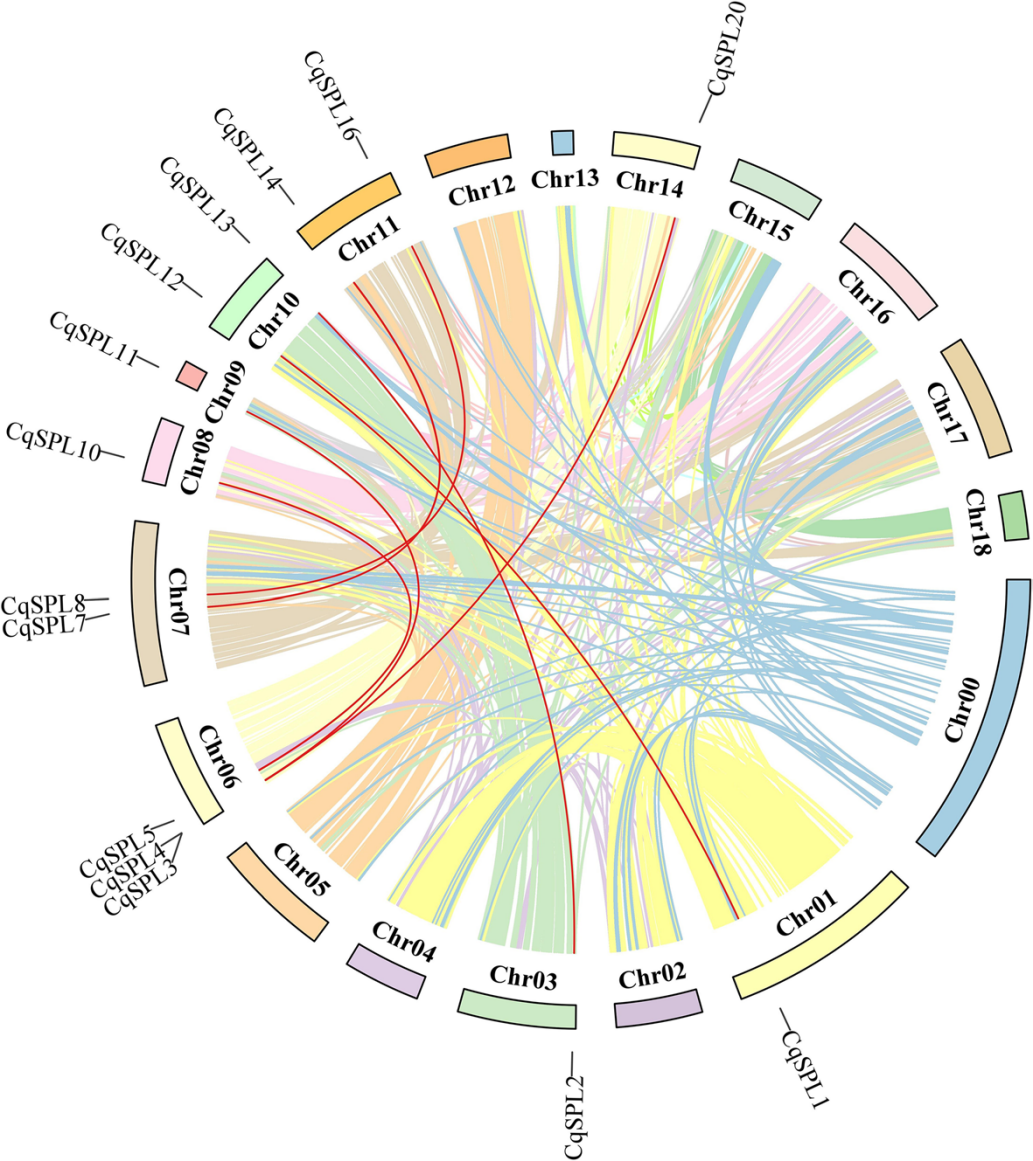
Analysis of interchromosomal fragment duplication of SPL genes in the quinoa genome. The colored lines represent all synthetic blocks and the red lines specifically indicate the duplicated pairs among the 23 CqSPL genes

Gene duplication events, which mainly include tandem repeat events and segmental duplications, play an essential role in gene amplification and the generation of new functions [41]. Tandem repeat events refer to the co-occurrence of two or more genes within a chromosomal region of ~ 200 kb [42]. Therefore, we performed a duplication event analysis of *CqSPL* genes to explore the evolutionary conservation of this gene family. We found that the quinoa genome exhibited seven pairs of duplicated fragments but no tandem repeat events (Fig. 5, Additional file 5: Table S4). The 14 paralogs that resulted from the seven pairs of duplicated fragments were denoted LG1-14, and their existence suggests an evolutionary relationship among the *CqSPL* genes. LG6 had the most *CqSPL*s (*n* = 3), followed by LG7, LG10, and LG14 (*n* = 2 each), while LG1, LG3, LG8, LG9, and LG14 each contained only one. As expected, all genes were linked within their subfamilies. Subfamily II had the most linked genes (e.g., four *SPL* genes), while subfamilies III, IV, V, VII, and VIII had two *SPL* genes each (Table S4). These results showed that some *CqSPL* genes may have been produced during fragment duplication and that these duplication events may have acted as a main evolutionary driver of the neofunctionalization of *CqSPL* genes.

### Evolutionary analysis of the CqSPL and SPL genes of different species

**Fig. 6:**
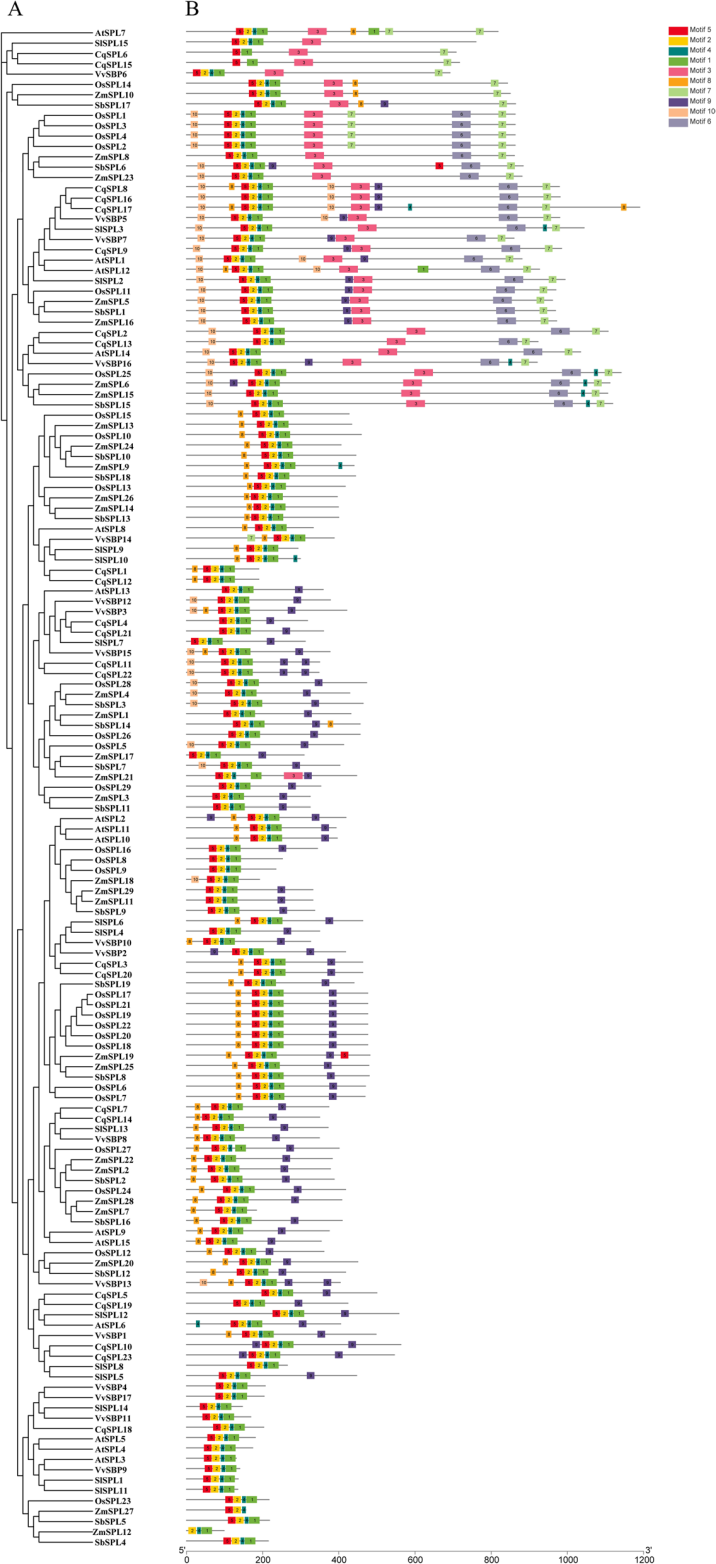
Phylogenetic relationships and motif compositions of SPL proteins of seven different plant species (C. quinoa, A. thaliana, S. lycopersicum, V. vinifera, S. bicolor, O. sativa, and Z. mays). **A** An unrooted phylogenetic tree was constructed using the neighbor-joining method as implemented by Geneious R11. **B** Distribution of the conserved motifs in SPL proteins. Ten differently colored boxes represent different motifs and their position in each SPL protein sequence (Table S2)

We selected three dicotyledonous plants (*Z. mays*, *O. sativa*, and *S. bicolor*) and three monocotyledonous plants (*A. thaliana*, *S. lycopersicum* and *V. vinifera*) for comparisons of *SPL* genes with *CqSPL*s. We used sequence data from the 23 *CqSPL*s and the *SPL* genes from the six other plants to construct a phylogenetic tree with ten conserved motifs (identified by the MEME web server) using the NJ method implemented in Geneious R11. The *CqSPL* genes exhibited an uneven distribution in the phylogenetic tree because genes from the same subfamily have the same motifs and therefore cluster together. Almost all *SPL* genes from the seven plants studied here contained motifs 1, 2, 4, and 5, but the first subfamily in quinoa (*CqSPL6* and *CqSPL15*) did not (Fig. 6, Additional file 2: Table S1). Subfamilies I and II contained the most diverse motifs, and motifs 10 and 7 were almost always distributed at the beginning and the end of the motif patterns, respectively. Meanwhile, we also found that motif 9 was always distributed at the end of the pattern in subfamilies III, IV, VII, and VIII. In conclusion, we found that *CqSPL* genes from groups I and III showed a high degree of homology with *SPL* gene clusters from *S. lycopersicum*. In contrast, most *SPL* genes in other groups clustered with *SPLs* from *A. thaliana*, *S. lycopersicum*, and *V. vinifera*, implying that they may be closely related and may therefore have similar functions.

**Fig. 7:**
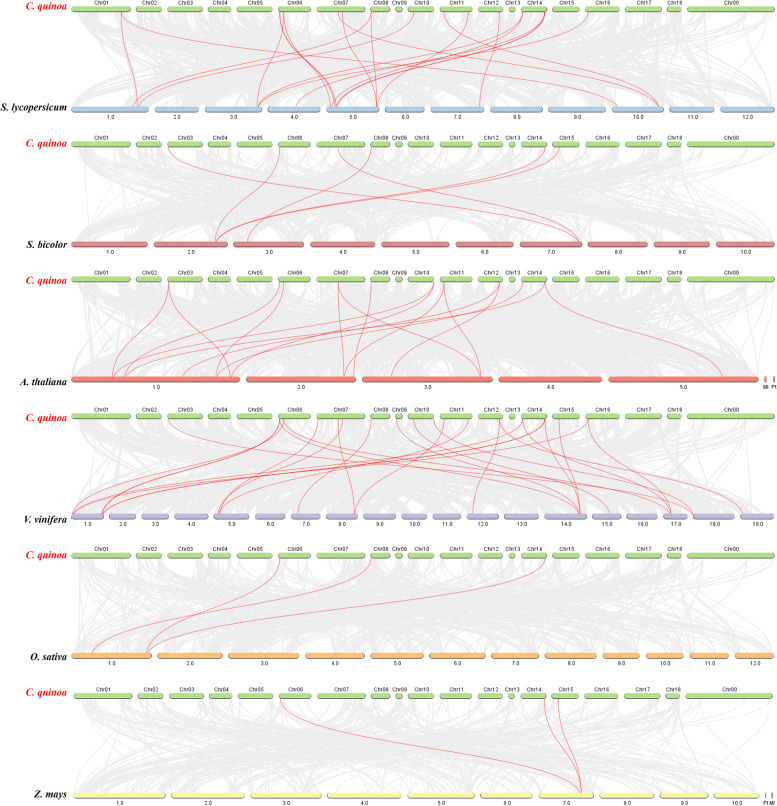
Analysis of SPL genes found in Chenopodium quinoa and in six representative plant species (A. thaliana, S. lycopersicum, V. vinifera, S. bicolor, O. sativa, and Z. mays). Gray lines in the background indicate neighboring blocks in the genomes of C. quinoa and other plants; red lines highlight syntenic C. quinoa SPL gene pairs

To further understand the phylogenetic relationships among the *SPL* genes, we constructed comparative syngeneic maps of quinoa and with the six other representative species. The 23 *CqSPL* genes showed collinear relationships with various *SPL*s found in *A. thaliana* (15), *S. lycopersicum* (15), *V. vinifera* (17), *S. bicolor* (19), *O. sativa* (29), and *Z. mays* (29) (Additional file 6: Table S5). The number of identified homologous pairs between quinoa and *Z. mays*, *O. sativa*, *S. bicolor*, *A. thaliana*, *S. lycopersicum*, and *V. vinifera* were 3, 3, 6, 16, 20, and 25, respectively (Fig. 7, Table S5).

We found at least one gene from each of the six plants that was collinear with an *CqSPL*, such as *CqSPL21*, which was collinear with Solyc05g015840/EER97011/AT5G50670.2/VIT_14s0068g01780/BGIOSGA005075/Zm00001d021056. This suggests that these orthologous genes were more highly conserved before divergence. We therefore speculate that they might have played an essential function in the evolution of the quinoa *SPL* gene family. Interestingly, some gene pairs collineating with 12 *CqSPL* genes were identified in *A. thaliana*, *S. lycopersicum*, and *V. vinifera* and not in *S. bicolor*, *O. sativa*, and *Z. mays*. This suggests that these orthologous pairs might have been formed via gene duplication events during the differentiation of dicotyledonous and monocotyledonous plants.

### Expression patterns of CqSPL genes in different plant organs

**Fig. 8:**
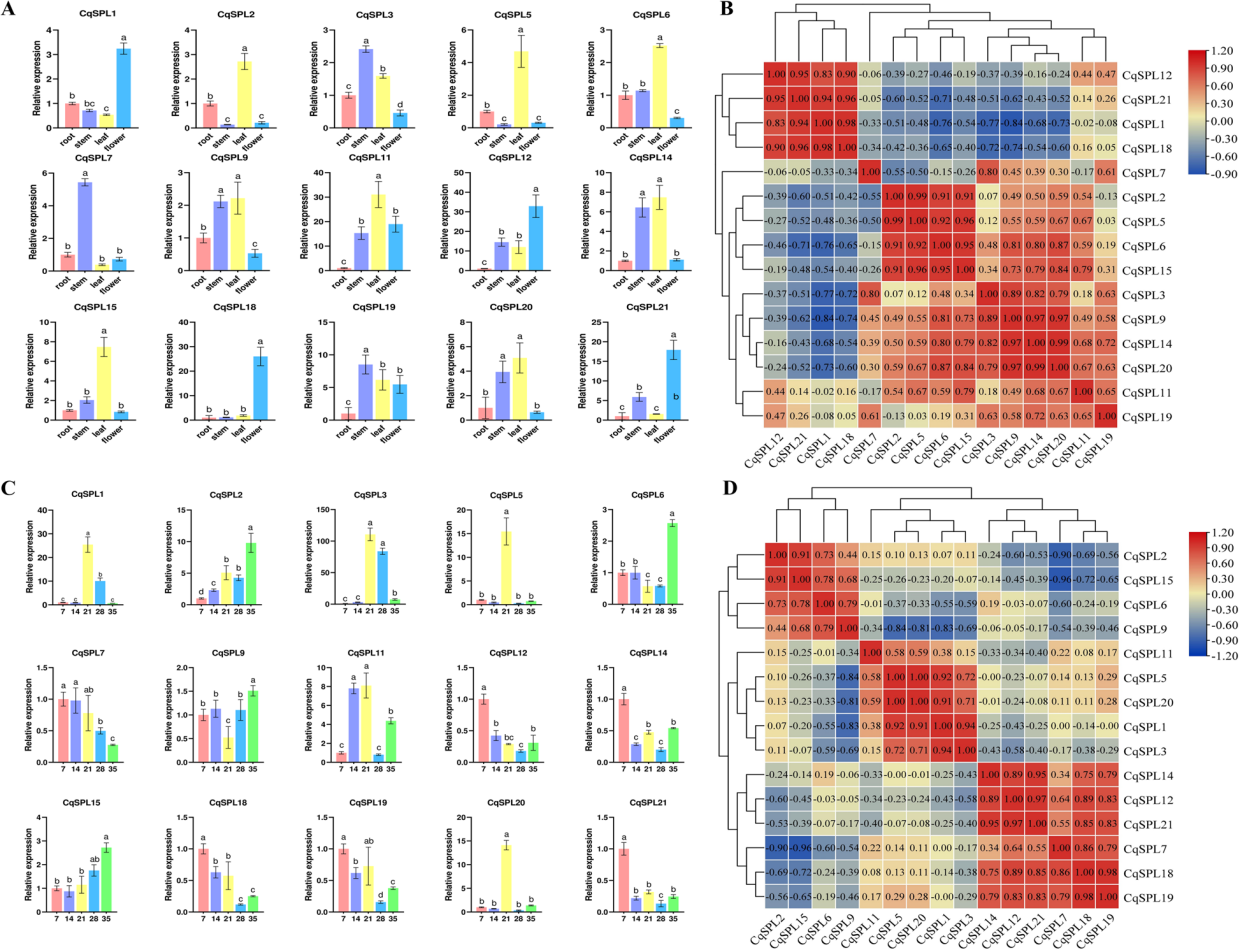
Gene expression of 15 CqSPL genes in various tissues and during fruit development. **A** Expression patterns of 15 CqSPL genes in flower, leaf, root, stem and fruit tissues as determined by qRT-PCR. Error bars represent standard error of three technical replicates. Lowercase letters indicate significant differences among treatment means (α = 0.05, LSD). **B** Positive numbers indicate positive correlations; negative numbers indicate negative correlations. Red numbers indicate statistically significant correlations (α = 0.05). **C** Expression patterns of 15 CqSPL genes at different developmental stages of quinoa fruit as determined by qRT-PCR (data shown are: 7 days post anthesis (DPA), 14 DPA, 21 DPA, 28 DPA, and 35 DPA). Error bars represent standard error of three technical replicates. Lowercase letters indicate significant differences among treatment means (α = 0.05, LSD). **D** Positive numbers indicate positive correlations; negative numbers indicate negative correlations. Red numbers indicate statistically significant correlations (α = 0.05)

The relative expression levels of 15 representative genes (selected from the eight subfamilies) was then analyzed in four organs (root, stem, leaf, and flower) by qRT-PCR to evaluate the potential function of *CqSPL* genes. We found that the *CqSPL* genes exhibited different expression patterns in roots, stems, leaves, and flowers, suggesting that these genes might play different regulatory roles. Three genes (*CqSPL3, CqSPL7*, and *CqSPL19*) showed the highest expression levels in stems, while eight genes (*CqSPL2, CqSPL5, CqSPL6, CqSPL9*, *CqSPL11, CqSPL14, CqSPL15*, and *CqSPL20*) showed the highest expression levels in leaves. Finally, *CqSPL1, CqSPL12, CqSPL18*, and *CqSPL20* were highly expressed in flowers (Fig. 8A) (*p* < 0.05). Most genes from the same subfamily exhibited similar expression patterns, suggesting that their functions might also be similar. In general, we found that *CqSPL* genes were expressed in root tissue to a lesser extent than in stems, leaves, or flowers. Therefore, we speculated that *SPL* genes might be more closely associated with stem, leaf, and flower development. The qRT-PCR analysis also showed differential expression patterns of *SPL* genes in different tissues and provides preliminary confirmation of the biological functions of *SPL* genes in quinoa.

Next, we reasoned that some *CqSPL*s might regulate fruit development of quinoa, thereby affecting its nutritional composition and development rate [3, 4]. We then analyzed the expression of 15 *CqSPL* genes at five different post anthesis intervals (i.e., 7 DPA, 14 DPA, 21 DPA, 28 DPA, and 35 DPA) to identify genes that may potentially regulate genes related to fruiting. Our results showed that most *CqSPL* genes exhibited different expression patterns at the five stages of fruit development. We found a significant increase in the expression of two genes (*CqSPL2* and *CqSPL15*) and a decrease in the expression of another two genes (*CqSPL7* and *CqSPL18*) in quinoa fruit. Interestingly, we also found that *CqSPL1*, *CqSPL3*, *CqSPL5*, *CqSPL11*, and *CqSPL20* showed the highest expression on day 21 of fruit development, while the expression of most *CqSPL* genes (i.e., *CqSPL5, CqSPL11, CqSPL12, CqSPL14, CqSPL18, CqSPL19*, *CqSPL19*, and *CqSPL20*) was the highest at 28 days (Fig. 8C) (*p* < 0.05). These findings also demonstrated that *SPL* genes play an essential role in fruit development, and provides a theoretical basis for studying the nutritional value of quinoa. Furthermore, we also observed notable correlations between patterns of *CqSPL* gene expression (Fig. 8). In general, we observed positive correlations between the expression levels of most *CqSPL* genes. However, we also found significant negative correlations between the expression levels of several *CqSPL* genes, such as *CqSPL6* with *CqSPL21*/*CqSPL1* and *CqSPL1* with *CqSPL9* (*p* < 0.05).

### Expression patterns of CqSPL genes under abiotic stress conditions

**Fig. 9:**
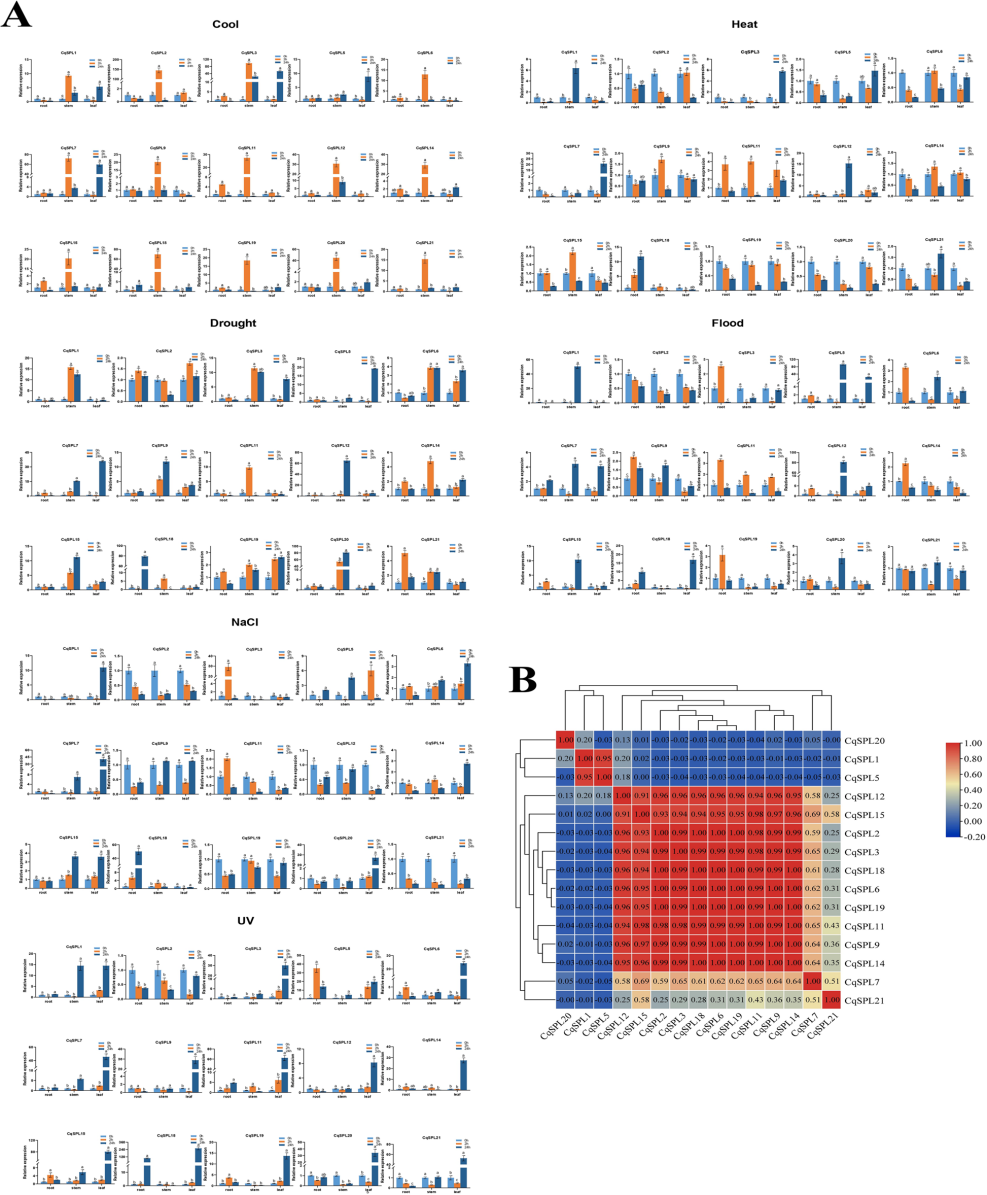
Expression analysis of 15 CqSPL genes in samples from root, stem, and leaf tissue of seedlings subjected to different abiotic stress treatments (i.e., UV radiation, flooding, PEG, NaCl, heat, and cold treatments). **A** Analysis of the relative expression of 15 CqSPL genes as determined by qRT-PCR. Error bars represent standard error of three technical replicates. Lowercase letters above the bar indicate statistically significant differences among means (α = 0.05, LSD). **B** Positive numbers indicate positive correlations; negative numbers indicate negative correlations. Red numbers indicate a statistically significant correlation (α = 0.05)

To determine whether different abiotic stresses affected the expression of *CqSPL* genes, we then evaluated the expression of 15 *CqSPL* genes in root, leaf, and stem tissue after subjecting plants to one of six abiotic stress treatments. Our results showed that some *CqSPL* genes were significantly up-regulated, while others were significantly downregulated, under different stress treatments. Most *CqSPL* genes also showed significant differences in expression levels among different tissues, and this effect often increased with treatment time, depending on the stress treatment [43]. For example, the expression of most *SPL* genes was up-regulated by cold stress treatment in stems, and the expression of *CqSPL11* and *CqSPL12* genes was initially up-regulated but later downregulated in roots, leaves, and stems. Moreover, in stems under flooding stress, *CqSPL1* and *CqSPL5* were significantly up-regulated, while *CqSPL2* was significantly downregulated. In general, most genes exhibited different patterns in plants subjected to different treatments and were significantly downregulated during the early phases of the treatments. *CqSPL1, CqSPL7, CqSPL5, CqSPL18*, and *CqSPL20* demonstrated similar expression patterns under different conditions. Moreover, we also found that in all tissue types many SPLs were up-regulated after prolonged treatment times, indicating that their expression can be rapidly inhibited by abiotic stress. However, the expression patterns of some *SPL*s, including *CqSPL2*, *Cq*SP*19,* and *CqSPL20*, showed the opposite trend. For example, their expression was up-regulated by heat stress but downregulated by cold stress in stem samples (Fig. 9) (*p* < 0.05). Notably, we found that *CqSPL1* was highly expressed in all plant tissues under all six stress treatments. Thus, it may be generally responsible for abiotic stress responses in quinoa.

The expression patterns of *CqSPL* genes showed instances of coordinated expressions in response to several abiotic stress treatments (Fig. 9B). Moreover, we observed positive correlations between the expression levels of most *CqSPL* genes. For example, nine genes (i.e., *CqSPL12, CqSPL15, CqSPL2, CqSPL3, CqSPL18, CqSPL6, CqSPL19, CqSPL11, CqSPL9,* and *CqSPL14*) were significantly positively correlated with each other, and *CqSPL1* and *CqSPL5* were also significantly positively correlated with each other. On the other hand, we also identified pairs of *CqSPL* genes (e.g., *CqSPL5* and *CqSPL20*) whose expression levels were significantly negatively correlated (*p* < 0.05).

## Discussion

### Structure of CqSPL genes and evolutionary analyses

Quinoa is a pseudocereal crop with a high nutritional value that originated from a genomic fusion between two diploid parent species (pale bulbous quinoa and lambda) [1]. Studies have shown that quinoa is rich in vitamins, polyphenols, flavonoids, saponins, and phytosterols, components that are known to provide health benefits [2]. *SPL*s, transcription factors related to inflorescence branching and grain development, have been shown to play important roles in the growth and development of quinoa [44, 45]. The rapid development of genome sequencing technologies has enabled the identification and characterization of *SPL* genes in many plants, including *O. sativa* [46], *A. thaliana* [47], *Z. maize* [19], *T. buckwheat* [25], *S. lycopersicum* [24], and *V. vinifera* [22]. However, to date no SPL genes have been identified in quinoa. Therefore, in this study we identified and performed a preliminary functional test of *SPL* gene family members in quinoa.

Here we identified 24 genes that encode SPL proteins with lengths ranging between 190 and 1190 amino acids (Figs. 1, 2 and additional file 1, 2: Figure S1, Table S1). A comparative genomic analysis of their gene structures revealed that the 24 *SPL* genes contained different numbers of introns, with a minimum of 2 and a maximum of 16. The *SPL* proteins examined here exhibited complex and variable structures that may be attributable to gene duplication events during evolution. In general, introns increase the length and the frequency of recombination between genes and modify their regulatory roles [48]. However, genes without introns may represent genes whose regulatory responses were conserved during evolution [49–52]. Our functional tests revealed that most *CqSPL* members rapidly responded to abiotic stress treatments, and those from the same subfamilies showed similar motifs and intron numbers and compositions. Thus, we speculate that they may share a common evolutionary origin and molecular function, and this information may be useful for predicting the functions of unknown proteins.

The 24 identified *CqSPL* genes were divided into eight subfamilies. Each of these subfamilies contained at least one *SPL* gene from *Arabidopsis* and quinoa, which further suggested their conservation during evolution and also indicate a possible biological function (Fig. 2). Gene amplification is the main generator of new functional genes during evolution, and gene amplification events can be divided into segmental duplication and tandem replication events [53]. Tandem duplication events occupy a larger proportion of plant genomes than segmental replication, and account for approximately 10% of the genes present in *Arabidopsis* and rice [54, 55]. We found more SPL proteins in quinoa than in *A. thaliana* (15), *V. vinifera* (17), and *S. lycopersicum* (15), which may indicate there could be more gene duplication events in the evolutionary history of quinoa than in these other plant species. Such events could lead to new functional genes that could help plants adapt to harsh environments [56]. We also found that the 23 *CqSPL* genes were unevenly distributed on the 18 chromosomes of quinoa (Fig. 4); moreover, our homology analysis showed no tandem duplicate gene pairs, but we did identify seven pairs of fragment duplicates (Fig. 5). The existence of homologous genes on different quinoa chromosomes might have facilitated the evolution and diversification of *CqSPL* genes, which are more numerous in quinoa than in other dicotyledons such as *A. thaliana*, *V. vinifera,* and *S. lycopersicum*.

Next, we examined the classification of *SPL* genes from quinoa and six other plant species into eight taxa. *CqSPL* genes from subfamilies I and III showed higher homology with *SPL* gene clusters found in *S. lycopersicum*, whereas most *SPL* genes in the other groups clustered with *A. thaliana*, *S. lycopersicum*, and *V. vinifera*. Notably, we identified at least one pair of collinear genes (i.e., *CqSPL21* and *Solyc05g015840*/ *EER97011*/*AT5G50670.2*/*VIT_14s0068g01780*/*BGIOSGA005075*/*Zm00001d021056*), which may provide a theoretical basis for understanding their ancestry. Moreover, an analysis of orthologous genes also illustrated that *CqSPL*s had many homologous gene pairs in the dicotyledons, which indicated a high degree of homology (Table S2). In addition, we found that the *SPL* genes contained ten unique motifs, and that different subfamilies exhibited similar motif patterns. The *SPL* genes in subfamily II contained almost all of these ten motifs. These results indicated that *CqSPL* genes are closely related to those found in other dicotyledons and may share a common ancestry.

### CqSPL expression patterns and functional prediction

Gene expression analysis is essential for providing clues for functional prediction [57]. This study explored the expression patterns of 15 representative genes in different tissues and at different developmental stages. Our results showed that almost all *SPL* genes were differentially expressed (i.e., showed more than a twofold difference) in different tissues in response to different abiotic stress treatments (*p* < 0.05). For instance, we found that all *SPL* genes were significantly up-regulated in stems and leaves in response to cold and UV treatments. This finding suggests that it may be possible to adapt quinoa for growth at high altitudes due to its potential cold tolerance and UV resistance [58]. We also found that the expression of *SPL* genes was significantly up-regulated in leaves and stems in response to all six abiotic stress treatments. However, we observed the highest expression of *SPL* genes in roots subjected to flooding treatment, suggesting that roots play a key functional role in plant responses to flooding stress (Fig. 9). Notably, *CqSPL1* was expressed in response to all six abiotic stress treatments, demonstrating that it may be a potential candidate gene for breeding tolerance to various abiotic factors in quinoa.

Previous studies have reported that *SPL* genes play an important role in flower and fruit development in many plant species [59–61]. Our findings also suggest that *SPL* genes may be involved in vegetative growth because these genes are highly expressed in stems and leaves in response to different stress treatments. Furthermore, Chao reported that *AtSPL1* and *AtSPL012* exhibited significant differences in *Arabidopsis* inflorescence development, and that overexpression of these genes enhanced inflorescence heat tolerance [62]. In present study, the *CqSPL9* gene, which is homologous to *AtSPL1* and *AtSPL012*, was found to be up-regulated in stems subjected to heat treatment. Thus, the structural similarities between homologous genes may be crucial for predicting gene function. In addition, Xu revealed that *AtSPL2*, *9*, *10*, *11*, *13*, and *15* [15] may promote floral meristematic tissue homogeneity and flower induction. These genes were members of three classes that are homologous to *CqSPL* subfamilies V (*CqSPL3* and *CqSPL20*), VII (*CqSPL4, CqSPL11*, *CqSPL21*, and *CqSPL22*), and VIII (*CqSPL7* and *CqSPL14*). Phylogenetic analysis showed that *AtSPL1* and *AtSPL12* were highly homologous with the *CqSPL*s found in subgroup II, including *CqSPL2, CqSPL8, CqSPL9, CqSPL13, CqSPL16*, and *CqSPL17.* In addition, *AtSPL13* was found to be homologous to *CqSPL* genes belonging to subfamily VII, which include *CqSPL4*, *CqSPL11*, *CqSPL21*, and *CqSPL22*. At the same time, we also found that *AtSPL9* and At*SPL15* were similar to *CqSPL7* and *CqSPL14* (Figs. 2 and 3, Table S1). Finally, our qRT-PCR and functional analysis showed that *SPL* genes were significantly up-regulated in different tissues, including leaves and stems, during inflorescence development (Fig. 8) which suggests a possible functional role [63]. We speculate that such an expression pattern might be due to complex protein interactions responsible for coordinating the expression of multiple genes via a network of feedback mechanisms [64].

## Conclusion

This study reports the identification of 23 putative *CqSPL* genes that were found to be unevenly distributed throughout the 18 chromosomes of the quinoa genome. Moreover, these 23 genes were classified into eight subfamilies, and the motifs and structures of *SPL* genes from the same family were similar, suggesting that they may share biological functions. Furthermore, fragments and tandem repeats were found to be the main drivers of neofunctionalization in the *CqSPL* gene family, but that fragment repeats may also have contributed to the evolution of quinoa *SPL* genes. Taken together, our results indicate that the *CqSPL* gene family plays a critical role in quinoa development and its response to various abiotic stresses. Moreover, this is the first study to report the identification and systematically analysis of *SPL* genes in quinoa.

## Methods

### Gene identification

Whole genome data for *C. quinoa* was downloaded from the Ensembl genome database (http://ensemblgenomes.org), and *SPL* family genes were identified using two BLAST approaches [65, 66]. In brief, all possible SPL proteins were identified using the BLASTp algorithm (score value ≥ 100, e value ≤ 1e-10) with the trihelix protein sequence of *Arabidopsis* used as the reference sequence. The obtained SPL protein sequences were then converted into a Hidden Markov Model (HMM) file format containing SPL domains [67, 68] and were then searched against the protein family (PFAM) database (http://pfam.sanger.ac.uk) using an HMM model cutoff value of 0.01 as implemented by HMMER3 (http://plants.ensembl.org/hmmer/index.html) [69]. The availability of *SPL* core sequences was confirmed using both PFAM and the SMART search tool (https://smart.embl.de). Thereafter, identified *SPL* genes were used as query terms to search for SPL proteins using BLASTp (https://blast.ncbi.nlm.nih.gov/Blast.cgi?PROGRAM=blastp&PAGE_TYPE=BlastSearch&LINK_LOC=blasthome). Protein identification and characterization was performed by comparing sequence length, isoelectric point (pI), molecular weight (MW), and subcellular localization using ExPasy.

### SPL gene structure

Multiple alignments of identified protein sequences were conducted using ClustalW (using default parameters) to check for similarity with the domain sequences of *A. thaliana* SPL proteins. Subsequently, the deduced amino acid sequences of the SPL domains from different subfamilies were manually annotated using GeneDoc and Mega7.0 [70]. The Gene Structure DiSPLay Server (http://gsds.cbi.pku.edu.cn) was then used to analyze the exon–intron structures of the putative *SPL* genes. Finally, full protein sequences were identified using MEME (http:/meme.nbcr.net/meme/intro.html), with an optimum motif width of 6–200 and a maximum motif number of 10.

### Chromosomal distribution and gene duplication events

All *CqSPL* genes were mapped to *C. quinoa* chromosomes and their distribution was visualized using Circos [71]. Next, the multiple collinearity scanning toolkit X (MCScanX) was run using default parameters to identify the replication events in the evolutionary history of each *CqSPL* gene. Finally, the degree of homology between *CqSPL* genes and *SPL* genes from six other plants (*S. bicolor*, *O. sativa*, *Z. mays*, *A. thaliana*, *S. lycopersicum*, and *V. vinifera*) was determined using the Dual Synteny Plotter implemented in TBtools (https://github.com/CJ-Chen/TBtools).

### Phylogenetic analysis and classification of the CqSPL gene family

Identified CqSPL proteins were clustered into groups based on the classification scheme used for *A. thaliana* SPL genes (AtSPLs). A neighbor-joining (NJ) tree was generated to identify clusters; this was implemented using the Jukes-Cantor model in MEGA 7.0 and Geneious R11 with the BLOSUM62 cost matrix. We then constructed a multi-species phylogenetic tree that included all SPL protein sequences from quinoa as well as six other plant species (*S. bicolor*, *O. sativa*, *Z. mays*, *A. thaliana*, *S. lycopersicum*, and *V. vinifera*). All protein sequences were downloaded from the UniProt database (https://www.uniprot.org).

### Plant materials, growth conditions, and different abiotic stress in C. quinoa

Quinoa seeds were provided by Guizhou University. To generate plants, seeds were first germinated in a petri dish lined with wet filter paper. After germination, seedlings were moved to a cultivation pot with nutrient soil, then placed under a plant light incubator for cultivation. The temperature setpoint was 25 °C. After six weeks of growth, we collected samples of leaves, roots, stems, grains, and flowers from five plants that showed similar growth features; these samples were snap-frozen in liquid nitrogen and stored at -80℃. The plants were then subjected to various abiotic stress treatments at the seedling stage (i.e., 21 days after germination) to determine how the expression patterns of *SPL* genes differed in response to different stress conditions. Stress treatments considered here included salt treatment (i.e., addition of 5% w/w sodium chloride), complete immersion of the plant in water, drought treatment (i.e., implemented by adding 30% PEG 6000), UV radiation (70 W/cm^2^, 220 V, 30 W), high temperature (40℃) treatment, and a low temperature (4℃) treatment. Five replicates were created for treatment and qRT-PCR analysis was performed on samples taken 2 h and 24 h post-treatment.

### Total RNA extraction, cDNA synthesis, and qRT-PCR analysis

RNA extraction was conducted using a plant RNA extraction kit (Vazyme Biotech, Shanghai, China). Next, cDNA libraries were constructed from 1 mg of each RNA sample via reverse transcription using 5 × HiScript® Reverse Transcriptase supplemented with a 4 × gDNA wiper solution for genomic DNA removal (Vazyme Biotech). We selected representative genes for expression analysis, which was conducted via qRT-PCR using primers designed by Beacon Designer 7 (Additional file 6: Table S5). We obtained data from three biological replicates for all qRT-PCR analyses. *ACTIN*, which is stably expressed in almost all plant tissues, served as an internal control, and the delta-delta Ct (2^−ΔΔCt^) method was used to calculate the relative gene expression levels of the samples [72].

### Statistical analysis

JMP 6.0 (SAS Institute) was used to perform analysis of variance (ANOVA) tests; multiple comparison tests of ANOVA results were performed using the least significant difference (LSD) method and the *p* < 0.05 and *p* < 0.01 significance levels. Finally, histograms were generated using Origin version 8.0 (OriginLab, Northampton, MA, USA).\

## Supplementary Information

**Additional file 1:** Supplementary **Figure. S1.** Alignment of multiple CqSPL and select SBP domain amino acid sequences.

**Additional file 2:** **Supplementary Table S1. **List of the 23 CqSPL genes identified in this study.

**Additional file 3:** **Supplementary Table S2.** Analysis and distribution of conserved motifs in Chenopodium quinoa  and other plants SPL proteins.

**Additional file 4: Supplementary Table S3. **The tandem duplication events of CqSPL genes. 

**Additional file 5:** **Supplementary Table S4.** The 7 pairs of segmental duplicates in C. quinoa SPL genes. 

**Additional file 6:** **Supplementary Table S5. **One-to-one orthologous relationships between Oryza sativa and Chenopodium quinoa.

**Additional file 7:** **Supplementary Table S6.** Primers of sequences.

**Additional file 8:** **Supplementary Table S7.** Cis-regulatory elements in the promoter region of SPL genes. 

## Data Availability

Whole genome sequence information for quinoa was obtained from the Ensembl genome website (http://ensemblgenomes.org). Quinoa seed material was provided by Yu Fan of Guizhou University. The datasets supporting the conclusions of this study are included in the article and in additional files.

## Abbreviations

SPL: SQUAMOSA promoter-binding protein-like
CqSPL: Chenopodium quinoa SPL
qRT-PCR: Quantitative real-time polymerase chain reaction
AtSPL: Arabidopsis thaliana SPL
HMM: Hidden Markov Model
pI: Isoelectric point
LG: Linkage group
DPA: Days post anthesis
