# Supplementary figures and images for: Genome-wide identification and expression analysis of the SPL transcription factor family and its response to abiotic stress in Quinoa (Chenopodium quinoa)
Source: BMC Genomics. 2022 Nov 25;23:773. doi: 10.1186/s12864-022-08977-9 (PMC9701020; doi:10.1186/s12864-022-08977-9)

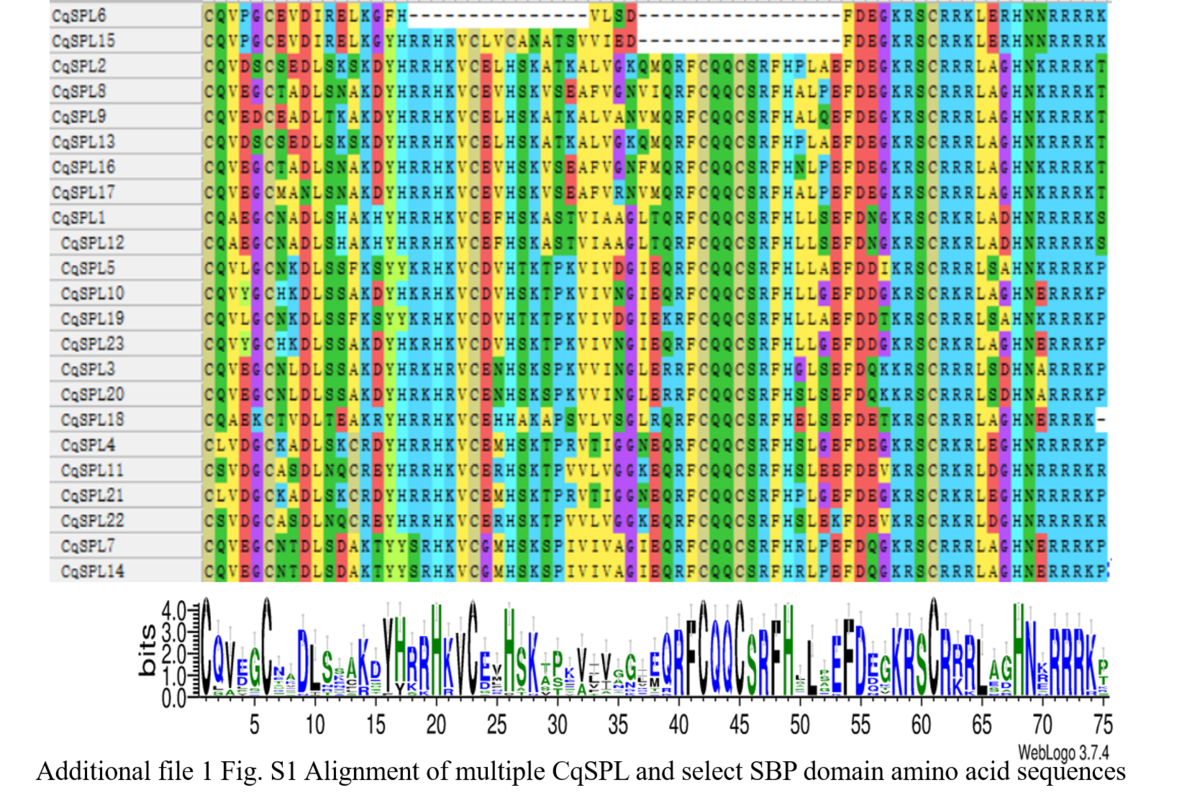

Supplement: Supplementary file 1 — Additional file 1: Supplementary Figure. S1. Alignment of multiple CqSPL and select SBP domain amino acid sequences. [file 12864_2022_8977_MOESM1_ESM.png]
